# Nano-Mole Scale Side-Chain Signal Assignment by ^1^H-Detected Protein Solid-State NMR by Ultra-Fast Magic-Angle Spinning and Stereo-Array Isotope Labeling

**DOI:** 10.1371/journal.pone.0122714

**Published:** 2015-04-09

**Authors:** Songlin Wang, Sudhakar Parthasarathy, Yusuke Nishiyama, Yuki Endo, Takahiro Nemoto, Kazuo Yamauchi, Tetsuo Asakura, Mitsuhiro Takeda, Tsutomu Terauchi, Masatsune Kainosho, Yoshitaka Ishii

**Affiliations:** 1 Department of Chemistry and University of Illinois at Chicago, Chicago, Illinois, United States of America; 2 JEOL RESONANCE Inc., Akishima, Tokyo, Japan; 3 RIKEN CLST-JEOL collaboration center, RIKEN, Yokohama, Kanagawa, Japan; 4 School of Science and Technology, Nazarbayev University, Astana, Kazakhstan; 5 Nuclear Magnetic Resonance Core Lab., King Abdullah University of Science and Technology, Thuwal, Saudi Arabia; 6 Department of Biotechnology, Tokyo University of Agriculture and Technology, Koganei, Tokyo, Japan; 7 Structural Biology Research Center, Graduate School of Science, Furocho, Chikusa-ku, Nagoya University, Nagoya, Japan 464–8601; 8 SAIL Technologies Co., Inc., Tsurumi-ku, Yokohama, Kanagawa, Japan; 9 Center for Priority Areas, Tokyo Metropolitan University, Tokyo, Japan; 10 Center for Structural Biology, University of Illinois at Chicago, Chicago, Illinois, United States of America; University of Pittsburgh School of Medicine, UNITED STATES

## Abstract

We present a general approach in ^1^H-detected ^13^C solid-state NMR (SSNMR) for side-chain signal assignments of 10-50 nmol quantities of proteins using a combination of a high magnetic field, ultra-fast magic-angle spinning (MAS) at ~80 kHz, and stereo-array-isotope-labeled (SAIL) proteins [Kainosho M. *et al*., Nature **440**, 52–57, 2006]. First, we demonstrate that ^1^H indirect detection improves the sensitivity and resolution of ^13^C SSNMR of SAIL proteins for side-chain assignments in the ultra-fast MAS condition. ^1^H-detected SSNMR was performed for micro-crystalline ubiquitin (~55 nmol or ~0.5mg) that was SAIL-labeled at seven isoleucine (Ile) residues. Sensitivity was dramatically improved by ^1^H-detected 2D ^1^H/^13^C SSNMR by factors of 5.4-9.7 and 2.1-5.0, respectively, over ^13^C-detected 2D ^1^H/^13^C SSNMR and 1D ^13^C CPMAS, demonstrating that 2D ^1^H-detected SSNMR offers not only additional resolution but also sensitivity advantage over 1D ^13^C detection for the first time. High ^1^H resolution for the SAIL-labeled side-chain residues offered reasonable resolution even in the 2D data. A ^1^H-detected 3D ^13^C/^13^C/^1^H experiment on SAIL-ubiquitin provided nearly complete ^1^H and ^13^C assignments for seven Ile residues only within ~2.5 h. The results demonstrate the feasibility of side-chain signal assignment in this approach for as little as 10 nmol of a protein sample within ~3 days. The approach is likely applicable to a variety of proteins of biological interest without any requirements of highly efficient protein expression systems.

## Introduction

^1^H indirect detection was introduced to ^13^C and ^15^N biomolecular high-resolution solid-state NMR (SSNMR) about a decade ago. [1–4] Despite its potential as a powerful tool to enhance sensitivity and resolution, ^1^H-detected SSNMR is not widely used due to limits on ^1^H resolution, even under fast MAS, and the lack of a demonstrated sensitivity advantage over more commonly used ^13^C detection. The recent introduction of ^1^H dilution by high-level deuteration and partial back-exchange of amide ^1^H (10–20%) has greatly improved the resolution of ^1^H SSNMR for biomolecules,[5, 6] offering a practical protocol for ^1^H indirect detection in protein SSNMR. However, the method is limited by a gross loss of ^1^H signals from amide sites (80–90%) due to extensive deuteration. ^1^H-detected ^13^C SSNMR for a fully protonated protein at very fast MAS (~40 kHz) has been used to obtain signal assignments for a model protein. [7] Nevertheless, this method is still hampered by relatively broad ^1^H line widths (0.5–1 ppm), a resolution that is insufficient even for small proteins. More importantly, it has been difficult to improve the sensitivity of 2D ^1^H indirect detection over that of standard 1D ^13^C direct-detected SSNMR. Recent studies described ^1^H indirect detection under ultra-fast MAS (UFMAS) at spinning frequencies of 60 kHz, resulting in resolved amide ^1^H resonances for fully deuterated proteins with fully back-exchanged amide proton[8, 9] or for undeuterated proteins. [10] Although those studies demonstrated the feasibility of main-chain sequential assignments for micro-crystalline samples, no strategy for assigning side-chain resonances by ^1^H-detected SSNMR has yet been developed, despite the fundamental importance of side chain structures and dynamics for protein functions. Equally importantly, no previous studies established advantage of ^1^H indirect detection method over traditional ^13^C direct detection for concurrent improvement in sensitivity and resolution by a quantitative analysis. Although some previous studies demonstrated sensitivity advantage of 2D ^1^H-detected SSNMR over 2D ^13^C-detected SSNMR,[7, 11, 12] it was difficult to achieve sensitivity advantage by ^1^H-detected (*N*+1)-dimensional SSNMR over a corresponding *N*-dimensional ^13^C-detected SSNMR scheme with an additional ^1^H dimension for higher resolution (*N* = 1, 2..). To overcome these problems, in this study, we propose the use of stereo-array isotope labeling (SAIL) as a highly effective labeling scheme suitable for side-chain signal assignments by ^1^H-detected protein SSNMR. The SAIL scheme was originally introduced to overcome the size limitation of biomolecular solution NMR by incorporating stereo-selective deuteration to achieve isolated ^1^H throughout all side chains of a protein.[13] Although ^1^H SSNMR was attempted for a SAIL amino acid (L-valine) under fast MAS at ~30 kHz,[14] resultant ^1^H line widths were still in a range of 0.5–0.7 ppm; the limited ^1^H resolution has hampered successful use of SAIL labeling for protein SSNMR. In addition, it is not trivial to determine whether sufficient sensitivity can be achieved for a SAIL-labeled protein sample under UFMAS conditions for limited sample quantity in a smaller MAS rotor and potentially much longer ^1^H *T*_1_ values due to deuteration. In this work, we demonstrate that a combination of UFMAS and SAIL selective deuteration significantly improves the sensitivity of ^1^H-detected 2D ^13^C SSNMR of biomolecules over 1D and 2D ^13^C direct detection. It is also discussed that this combination offers extremely sensitive means of biomolecular SSNMR for side-chain assignments with resolution enhanced ^1^H signals having line widths of 0.1–0.2 ppm.

## Results and Discussion

**Fig 1:**
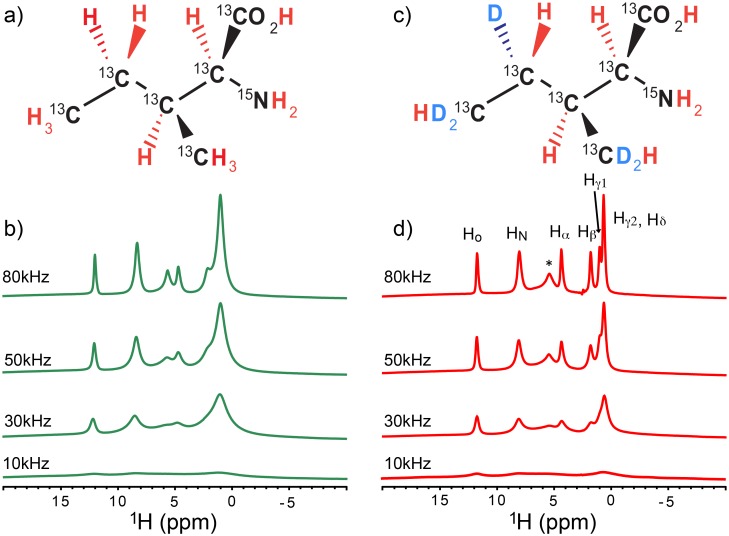
Spinning-speed dependence of 1H MAS spectra of fully protonated and SAIL isoleucine samples. (a, b) Chemical structures of (a) uniformly ^13^C- and ^15^N-labeled (UL) Ile and (b) SAIL-Ile. (c, d) Spinning-speed dependence of ^1^H MAS SSNMR spectra of (c) UL-Ile and (d) SAIL-Ile. The peak at 4.8 ppm (*) is likely due to HCl salts.[17, 18] No window functions were applied.

First, in experiments on amino-acid samples, we investigated whether the combined use of SAIL labeling with UFMAS in a high magnetic field of 17.62 T (^1^H frequency of 750.15 MHz) could improve the resolution of ^1^H SSNMR resolution. Fig 1(a, b) shows chemical structures and labeling schemes for (a) uniformly ^13^C- and ^15^N-labeled isoleucine (UL-Ile) and (b) SAIL-isoleucine (SAIL-Ile). Unlike random deuteration, in a SAIL scheme, all the protonated ^13^C groups are connected to a single ^1^H species. This feature allows preparation of strong ^13^C polarization for all ^13^C species via efficient double-quantum cross-polarization from directly bonded ^1^H nuclei.[15] More importantly, isolated ^1^H spins allow us to achieve very high resolution without the effects of strong ^1^H–^1^H dipolar couplings. Fig 1(c, d) shows the spinning-speed dependence of ^1^H MAS SSNMR of (c) UL-Ile and (d) SAIL-Ile, with signal assignments provided in (d). Significant improvement in resolution and sensitivity was obtained at higher spinning speeds (ν_R_). Resolution enhancements of 2–3-fold were observed at ν_R_ = 80 kHz in (d) relative to ν_R_ = 30 kHz, which was used in previous studies of ^1^H SSNMR on SAIL-valine.[14] Clearly, SAIL-Ile provided much higher resolution in Fig 1d (ν_R_ = 80 kHz) relative to the corresponding spectrum for UL-Ile in Fig 1c. In particular, for H_β_, H_γ_, and H_δ_ groups, the spectrum for SAIL-Ile exhibits a dramatic improvement in resolution (by a factor of 3–4) relative to the resolution for UL-Ile, with ^1^H widths of 0.21–0.25 ppm at 80 kHz. This narrowing can be attributed to the isolation of ^1^H in methylene and methyl groups by stereo-specific deuteration in the SAIL scheme.[13, 16] Much broader ^1^H line widths (0.7–1.0 ppm) were observed for these groups in UL-Ile (Fig 1c). These observations confirm that UFMAS itself is still not sufficient to remove broadening due to strong ^1^H–^1^H dipolar couplings within the CH_2_ and CH_3_ groups, even at ν_R_ of ~80 kHz. The combination of SAIL and UFMAS also exhibited modest narrowing (15–20%) in the line widths of O–H (0.22 ppm) and ^1^H_α_ (0.26 ppm) (Fig 1d). We also confirmed the excellent ^1^H resolution at ν_R_ of ~80 kHz for SAIL Thr (Fig A in S1 File).

The sensitivity enhancement factor (*ξ*) of ^1^H indirect detection over direct detection of a dilute X nuclei depends on the line width in the ^1^H dimension *W*_H_, the apodization, and the efficiency of polarization transfer (*f*) from X to ^1^H used for ^1^H detection, as shown in Eq (1),[1, 2]

$$\xi =\frac{f}{\sqrt{\alpha }}{\left(\frac{\gamma_{\text{H}}}{\gamma_{\text{X}}}\right)}^{3/2}{\left(\frac{W_{\text{X}}}{W_{\text{H}}}\right)}^{1/2}{\left(\frac{Q_{\text{X}}}{Q_{\text{H}}}\right)}^{1/2},$$ (1)

where γ_H_ and γ_X_ represent the gyromagnetic ratios of the nuclei H and X, W_H_ and W_X_ are the line widths observed for ^1^H and X nuclei, and Q_H_ and Q_X_ are the quality factors for the sample coil for ^1^H and X detection, respectively. The factor *α* is 1 for a comparison of 2D ^1^H-detected X/^1^H correlation SSNMR with 2D X-detected X/^1^H correlation. For a comparison of 2D ^1^H-detected X/^1^H correlation with 1D ^13^C CPMAS, the *α* value becomes 2π assuming apodization with matched window functions (see SI about the details).[1, 2] Thus, there is a ~2.5-fold difference (i.e.,$\sqrt{2\pi }$) between the *ξ* values of the 1D and 2D direct X-detection experiments. In biomolecular SSNMR, it is typical that no or minimal line broadening is applied for higher spectral resolution. When no window functions are applied in the ^1^H dimension, *α* ~ 2*π* ^2^ for the comparison with 1D ^13^C SSNMR; thus, this suggests a 4.4-fold difference (i.e.,$\sqrt{\text{2}}\pi$) between the *ξ* values for 1D and 2D (see SI about the details). Compared with the ^1^H resolution for UL-Ile at ν_R_ = 40 kHz, which was previously used in ^1^H-detected protein SSNMR, [7] the ^1^H resolution for CHD and CHD_2_ groups improved as much as 5–6-fold for the SAIL sample at ν_R_ = 80 kHz. We confirmed that the transfer efficiency *f* by ^1^H–^13^C double-quantum CP at ν_R_ = 80 kHz was comparable to the CP efficiency at ν_R_ = 20–40 kHz for SAIL-Ile. Thus, this new combination of SAIL and UFMAS offers opportunities to dramatically improve the sensitivity and resolution of ^1^H detection.

**Fig 2:**
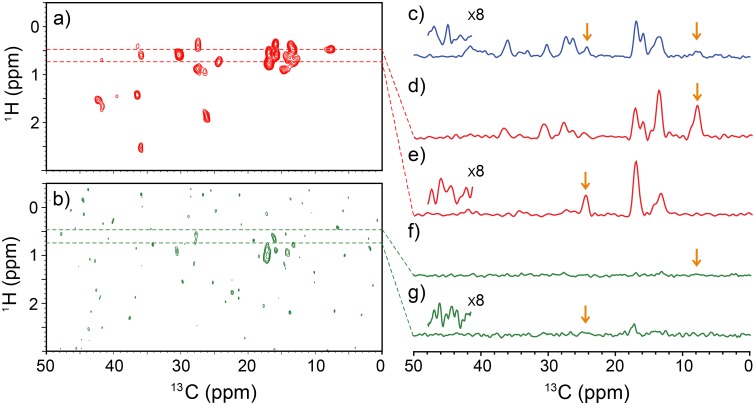
A comparison of 1H-detected and 13C-detected 2D and 1D SSNMR spectra of SAIL ubiquitin. (a) ^1^H-detected and (b) ^13^C-detected 2D ^13^C/^1^H spectra and (c) a 1D ^13^C CP-MAS spectrum of SAIL-Ubq (~0.5 mg) at MAS 80 kHz. (d–g) 1D slices from ^1^H shifts of (d, f) 0.43 ppm and (e, g) 0.69 ppm from (d, f) ^1^H-detected and (e, g) ^13^C-detected experiments. All spectra were processed with 45°- and 60°-shifted sine-bell window functions in the ^1^H and ^13^C dimensions, respectively. The ^1^H and ^13^C line widths were 0.10–0.22 ppm and 0.66–0.94 ppm, respectively, in the absence of any window functions. The insets in (c, e, and g) show the magnified noise regions. Each spectrum in (a–c) was collected within ~5 min. The pulse sequences used for (a) and (b) are shown in Fig B and Fig C in S1 File.

Next, we explored the possibility of improving the sensitivity and resolution of ^13^C SSNMR for SAIL proteins. As a suitable benchmark, we selected a micro-crystalline sample of ubiquitin (Ubq) that was selectively labeled with SAIL-Ile to compare the resolution with that of the amino acid data. Because there are as many as seven Ile residues in ubiquitin, the system is also suited for investigating improvements in the resolution of ^1^H detection. Another major challenge is the limited amount of sample used in these experiments. Because the engineering needs for UFMAS at 80 kHz limit the sample volume to only ~1 μL, it was difficult to achieve sufficient sensitivity in multi-dimensional ^13^C protein SSNMR, even in a high magnetic field. Fig 2a and 2b shows (a) ^1^H-detected and (b) ^13^C-detected 2D ^13^C/^1^H correlation spectra of the SAIL-Ubq sample. In the ^1^H-detected 2D spectrum, the sensitivity was dramatically improved (by a factor of 5.4–9.7) (Fig 2a) relative to the ^13^C-detected 2D data (b), as shown from the comparison of the slices corresponding to the peaks indicated by arrows (d–g). The factors were confirmed with the data collected from additional scans for (b) (see Table A in S1 File). As a result of this significant improvement, the 2D spectrum in Fig 2a was obtained after only 5 min, using sub-milligram quantities of protein sample (~0.5 mg or ~55 nmol, excluding H_2_O). By contrast, the corresponding ^13^C-detected 2D spectrum in Fig 2b had a much lower signal-to-noise ratio. Because of the excellent ^1^H resolution, most of the resonances are well separated in the ^1^H-detected 2D spectrum in Fig 2a in contrast to the significant signal overlap observed in the 1D ^13^C SSNMR in (c). It should be noted that the backbone ^13^Cα signals and some of the ^13^Cβ signals were weak because the protein was expressed in a D_2_O medium and, consequently, ^1^Hα and some ^1^Hβ were replaced by ^2^H; this issue can be overcome by expressing the protein in a cell-free system. It is also noteworthy that only 7.5 mg of SAIL-Ile was needed for preparing the sample at high labeling efficiency (~90%) from an E. coli cell culture of 0.5 L. The ^1^H line widths were 0.14–0.25 ppm and 0.10–0.22 ppm, respectively, with and without window functions. Clearly, our approach has opened an avenue for micro-SSNMR analysis of a protein sample in a minimal experimental time.

The sensitivity enhancement factors by ^1^H-detected data in (d, e) relative to 1D ^13^C CPMAS in (c) for resolved ^13^CHD signals at 24.5 ppm and ^13^CHD_2_ signals at 7.9 ppm (orange arrows) were 2.1 and 5.0, respectively. These factors are slightly greater than the theoretical values 1.5–2.7, which were obtained by multiplying $\sqrt{1.5}/(\sqrt{2}\pi )$ by the experimental *ξ* values for the ^13^C-detected 2D experiment, where the factor $1/\sqrt{2}\pi$ came from the sensitivity difference between 2D and 1D experiments without window functions (see SI). The modest gain by a factor of $\sqrt{1.5}$ (or ~1.2) was expected from “time saving” due to a linear prediction, (LP) which was employed to extend the indirect time-domain signals by 1.5 fold although an apparent S/N ratio with LP may be influenced by other issues such as additional “noise” for prediction artifacts. We also experimentally confirmed similar sensitivity improvement factors *ξ* over 1D CPMAS (*ξ* = 1.3–2.0) and ^13^C-detected 2D correlation (*ξ* = 3.9–10.1) for SAIL-Ile (see Fig E and Table B in S1 File). To the best of our knowledge, this is the first demonstration that ^1^H-detected 2D ^1^H/^13^C correlation SSNMR for a protein sample is significantly more sensitive than 1D ^13^C direct detection.

**Fig 3:**
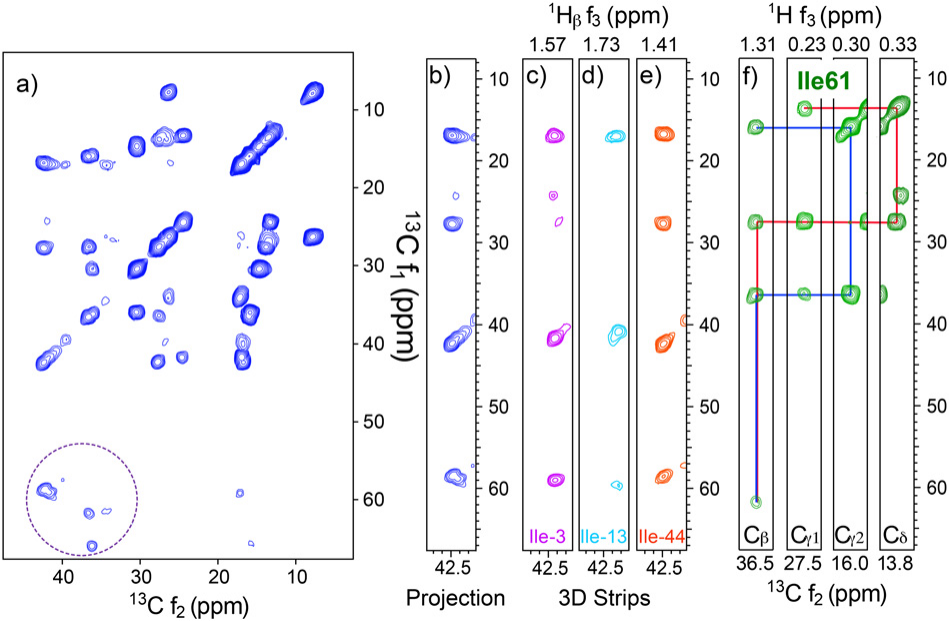
Resolution and side-chain assignments from 3D ^13^C/^13^C/^1^H SSNMR of SAIL-Ubq. (a, b) 2D ^13^C/^13^C 2D projection spectra from a ^1^H-detected 3D ^13^C/^13^C/^1^H SSNMR of SAIL-Ubq at MAS 80 kHz. All the peaks including minor ones in (a) are attributed to intra-residue cross peaks within the Ile residues. (c–e) Representative 2D ^13^C/^13^C slices corresponding to ^1^H chemical shifts of (c) 1.57 ppm, (d) 1.73 ppm, and (e) 1.41 ppm. The data show clear separation of signals for (c) Ile-3, (d) Ile-13, and (e) Ile-44 by ^1^H shifts. The spectrum was processed with 45°- and 60°-shifted sine-bell window functions in the ^1^H and ^13^C dimensions, respectively. (f) ^13^C/^1^H assignments for Ile-61 from the 3D data. The pulse sequence is listed in Fig D in S1 File.

The results described above suggest that most standard ^13^C-detected 2D and 3D SSNMR involving side-chain signals can be replaced by ^1^H-detected 3D and 4D SSNMR, respectively, with significantly enhanced *resolution and sensitivity*. To test this possibility, we performed ^1^H-detected 3D ^13^C/^13^C/^1^H correlation SSNMR on the SAIL-Ubq sample. Fig 3 shows (a,b) a 2D ^13^C/^13^C projection of the 3D data and (c–e) strip plots of ^13^C/^13^C 2D slices corresponding to ^1^H chemical shifts of (c) 1.57 ppm, (d) 1.73 ppm, and (e) 1.41 ppm. The 3D spectrum in Fig 3 was obtained in 2.5 h, despite the deuteration of C_α_ and partial deuteration of C_β_. We did not attempt a 3D experiment by ^13^C detection, as it would have taken up to 10 days. All the ^13^C resonances for the seven Ile residues are observed in Fig 3a, including those for nearly fully deuterated ^13^C_α_ (dotted circle). These ^13^C_α_ resonances were detected in the *t*_1_ period by polarization transfer from remote ^1^H, correlation to ^13^C_β_ in *t*_2_, and final detection at ^1^H_β_ in the *t*_3_ period. In the 2D ^13^C/^13^C projection, the signal overlap could not be completely eliminated. For example, the three signals for Ile-3, Ile-13, and Ile-44 are nearly overlapping at (*f*_1_, *f*_2_) ~ (59 ppm, 42 ppm) in the projection shown in Fig 3b. However, in 2D slices at three different ^1^H shifts (Fig 3c–3e), these overlapped peaks are clearly separated by well dispersed ^1^H_β_ shifts. All the side-chain ^1^H and ^13^C resonances of Ile, except for ^1^H_γ1_ of Ile-13, were assigned with excellent resolution (Table C in S1 File), as shown for an example of Ile-61 in Fig 3f. Although the ^13^C_α_ and ^13^C_β_ signals were assigned here to specific residues based on previous ^13^C-detected SSNMR studies,[19, 20] it is possible to connect side-chain resonances to main-chain and ^13^C_β_ resonances, which can be assigned from ^1^H-detected SSNMR for a uniformly ^13^C- and ^15^N-labeled protein as previously discussed. [10] It should be noted that the main-chain assignment strategy by ^1^H-detection [10] is likely to be applicable to uniformly SAIL-labeled proteins. In that case, spectral assignments for the main chain as well as side chains could be obtained by the ^1^H-detection method on the uniformly SAIL-labeled protein. Alternatively, site-directed mutagenesis can be used for assignments of some specific residues. Thus, the ^1^H-detected high-field SSNMR approach using SAIL-labeled protein and UFMAS is highly effective for side-chain assignments. The data suggest that side-chain assignments by 3D SSNMR analysis can be obtained from 10 nmol (or ~90 μg for Ubq) within only ~3 days in our approach.

## Conclusion

In this work, we discussed a general approach to achieve side-chain spectral assignments of SAIL-labeled proteins by ^1^H-detected protein SSNMR approaches. This work raises new prospects for high-field protein SSNMR in two areas. First, we presented the advantage of ^1^H-detected high-field SSNMR by demonstrating feasibility of side-chain assignments from 10–50 nmol of SAIL-labeled proteins under UFMAS at 80 kHz. To date no approach has been able to achieve efficient side-chain assignments through ^1^H-detected biomolecular SSNMR. Our data clearly demonstrate that, using this approach, heavily overlapped ^13^C side-chain signals can be resolved by ^1^H shifts for all seven Ile residues in SAIL-labeled ubiquitin with minimal sample requirements. Unlike methyl-selective labeling,[21] ^1^H-detected SSNMR for SAIL-labeled proteins offers ^13^C–^13^C connectivities and high-resolution ^1^H SSNMR for stereo-selectively labeled CHD groups for side-chain assignments. The method can be applied to structural analyses of a variety of proteins that are either selectively labeled with a set of different SAIL amino acids or uniformly labeled with SAIL amino acids. Although preparation of a SAIL-labeled protein in the large quantities required for conventional SSNMR experiments (0.5–1 μmol) is cost-prohibitive, the minimal sample requirement (10–50 nmol) of our approach makes such an approach very practical. The demonstrated reduction of sample requirements will make it feasible to implement even more advanced isotope labeling schemes or multiple sets of differently SAIL-labeled samples for future biomolecular SSNMR.

Secondly, we experimentally demonstrated that ^1^H indirect detection in 2D–3D SSNMR experiments on the SAIL-labeled protein notably improved sensitivity as well as additional ^1^H spectral resolution, over traditional ^13^C-direct detection in the corresponding 1D–2D experiments. This suggests that in our approach combining uses of UFMAS at 80 kHz and SAIL proteins, many of traditional 2D-3D ^13^C-detected experiments can be replaced by ^1^H-detected 3D-4D experiments. As discussed above, in most of previous studies the sensitivity advantage of ^1^H indirect detected SSNMR was described for less useful ^15^N SSNMR, rather than for ^13^C SSNMR, which is 4-fold more sensitive. In this work, we quantitatively demonstrated the sensitivity and resolution advantage of this method over traditional ^13^C-detected SSNMR in a high field (17.6 T), which is now widely available to the scientific community. We reported up to 10-fold sensitivity improvements by ^1^H detection and UFMAS at 80 kHz when a ^1^H-detected 2D–3D experiment is compared with an equivalent ^13^C-detected 2D–3D experiment. It will be feasible to reach the detection limit of a few nmol or sub-nmol of SAIL-labeled proteins by further sensitivity enhancement using paramagnetic doping[19, 22], modified polarization transfer schemes,[23], non-uniform sampling[24], and even faster MAS.[15, 25, 26] This approach is likely to applicable to a variety of micro/nano-crystalline proteins in order to drastically speed up side-chain spectral and structural analysis. Although it is outside the scope of this study, a similar approach using ^1^H- or ^19^F-detection should be possible for amyloid aggregates with reasonable structural homogeneity[27–33] and bioinorganic samples.[34, 35]

## Materials and Methods

The SAIL-Ile and UL-Ile used for these experiments were recrystallized in 20% DCl in D_2_O[17] before packing into a MAS rotor. SAIL-Ubq samples were expressed in *E*. *coli* BL21 cells harboring a plasmid containing the chlorella Ubq gene, cultured in D_2_O/M9 medium supplemented with SAIL-Ile, as described in the SI. The sample was crystallized by dissolving 2 mg of the lyophilized powder in 160 μL of citrate buffer in D_2_O and then precipitating with 240 μL d_12_-MPD (2-methyl-2,4-pentanediol).[20] Further details about the sample preparation are discussed in the SI.

All SSNMR experiments were performed on a Bruker Avance III 750MHz spectrometer at the UIC Center for Structural Biology using a JEOL 1 mm ^1^H/^13^C/^15^N/^2^H quad-resonance MAS probe. All the ^13^C–^1^H polarization transfers in this work were performed using adiabatic double-quantum cross-polarization (DQ-CP) schemes[19, 36], with the sum of the rf field strengths for *I* and *S* hetero-nuclear spins matched to *ω*_R_ (i.e., *ω*_1I_ + *ω*_1S_ = *ω*_R_) so that sample heating was minimized at high repetition rates by the low-power CP scheme.[10, 15] For ^1^H decoupling, a low-power decoupling scheme[37] with SPINAL-64[38] at 10 kHz was applied. For ^13^C, ^15^N, and ^2^H decoupling, a WALTZ-16 scheme was used at RF field strengths of 10, 2, and 5 kHz, respectively. All the multi-dimensional NMR data were processed using the nmrPipe software.[39] Unless stated otherwise, all indirect time-domain signals in the 2D and 3D data were extended to 1.5-fold and 2-fold by linear prediction, respectively. The multi-dimensional SSNMR data were apodized with 45°- and 60°-shifted sine-bell window functions in the ^1^H and ^13^C dimensions, respectively, to balance sensitivity and resolution. For the SAIL-Ile and Ubq samples, ^13^C decoupling was applied during the ^1^H detection/evolution periods, whereas the ^1^H and ^2^H decoupling sequences were applied during the ^13^C detection/evolution periods. For the SAIL samples, the sample temperature under UFMAS at 80 kHz was kept at ~37°C using a FTS cooler unit with N_2_ gas at ˗12°C.

The ^1^H NMR spectra in Fig 1 were collected with π/2-pulse direct excitation with WALTZ-16 ^13^C decoupling [3] at an RF field strength of 10 kHz with a recycle delay of 10 s. The details of the pulse sequences used for Figs 2 and 3 are listed in the SI (Figs B–D in S1 File). For the data in Fig 2, ^13^C polarization was prepared by DQ-CP transfer with a constant ^13^C RF field strength of ~2ν_R_/5 and a downward-ramped ^1^H RF field with an average strength of ~3ν_R_/5, using a contact time of 1.5 ms. Similar conditions, but with an upward ^1^H ramp, were used to transfer ^13^C polarization back to ^1^H for the ^1^H detection experiments in Fig 2a. The maximum *t*_1_ and *t*_2_ periods for Fig 2(a) and 2(b) were 5 ms and 10 ms, respectively. The recycle delay was set to 0.54 s for SAIL-Ubq, which had relatively short ^1^H *T*_1_ values (~0.25 s). For data processing, the indirect time-domain data along the *t*_1_ period were extended to 10 ms by linear prediction.

The 3D data in Fig 3 were obtained with a recycle delay of 0.52 s using the DQ—CP scheme (Fig C in S1 File) used for Fig 2a. After the *t*_1_ period for ^13^C evolution, the fpRFDR sequence[40] was employed for ^13^C–^13^C mixing. Subsequently, the ^13^C signal was recorded in the *t*_2_ period. Then, the ^1^H signal was acquired after DQ—CP transfer of the ^13^C polarization to ^1^H spins with a contact time of 0.5 ms. The signals were collected with maximum *t*_1_ and *t*_2_ periods of 2.4 ms and an acquisition period (*t*_3_) of 10.2 ms. The relatively short *t*_1_ and *t*_2_ periods were employed to optimize the sensitivity for the detection of weak deuterated ^13^C_α_ signals. The *t*_1_ and *t*_2_ data were extended to 3.6 ms by linear prediction and was processed as discussed above for Fig 2.

## Supporting Information

S1 File Fig. A, Spinning speed dependence of ^1^H MAS SSNMR of SAIL-threonine (SAIL-Thr) with its chemical structure and labeling scheme. The ^1^H NMR spectra were obtained using WALTZ-16 ^13^C decoupling with an RF field strength of 10 kHz. All the spectra were obtained with 2 scans with a pulse delay of 15 s, and the data were processed without any window function. The ^1^H line widths for H_α_, H_β_, H_γ_ OH/NH are 0.22 ppm, 0.22 ppm, 0.24 ppm, 0.20 ppm, respectively. **Fig. B, A pulse sequence for** ^**1**^ **H-detected 2D** ^**1**^ **H/** ^**13**^ **C chemical-shift correlation spectroscopy used for Fig 2a**. In this sequence, ^13^C spin polarization was prepared with adiabatic double-quantum cross polarization (DQ-CP) using an amplitude-modulated shaped pulse with a downward tangential ramp for the ^1^H channel and a rectangular pulse for the ^13^C channel. The ^1^H RF field strength was swept from 66.0 kHz to 26.4 kHz with the average rf field set at 46.2 kHz (~3ν_R_/5) while the ^13^C RF field amplitude was kept constant at 32.0 kHz (~2ν_R_/5). The contact time of the first CP was 1.5ms. During the *t*_1_ period, SPINAL-64 ^1^H decoupling[38] and WALTZ-16 ^2^H decoupling were applied with RF field strengths of 10 kHz and 5 kHz, respectively. The *t*_1_ period was incremented up to 5.1 ms with an increment of 37 μs. After the *t*_1_ period, a pair of π/2-pulses were applied as a Z-filter in order to select the real or imaginary component of the ^13^C polarization, which was transferred back to ^1^H spins with the second adiabatic CP using a reversed upward tangential ramp for the ^1^H channel and the same rectangular pulse for the ^13^C channel. The contact time of the second CP was 1.5 ms. During the acquisition (*t*_2_) period of 10.2 ms, ^1^H signals were acquired with dwell times of 5 μs under ^13^C decoupling using WALTZ-16 sequence[41] with an RF field strength of 10 kHz. The phase cycles for the pulse sequence were as follows: *ϕ*_1_ = y; *ϕ*_2_ = x; *ϕ*_3_ = x, x, -x, -x; *ϕ*_4_ = y, y, y, y, -y, -y, -y, -y; *ϕ*_5_ = x; *ϕ*_6_ = y, -y; *ϕ*_7_ = y; *ϕ*_8_ = x, -x, -x, x, -x, x, x, -x. The phase *ϕ*_3_ and the receiver phase were incremented along the *t*_1_ points using the States-TPPI data collection mode. **Fig. C, A pulse sequence used for** ^**13**^ **C-detected 2D** ^**1**^ **H/** ^**13**^ **C chemical-shift correlation spectroscopy in Fig 2b**. A pulse sequence used for ^13^C-detected 2D ^1^H/^13^C chemical-shift correlation spectroscopy in Fig 2b. After excitation by a π/2-pulse, ^1^H spin polarization evolved under ^1^H chemical-shift interactions during the *t*_1_ period under WALTZ-16 ^13^C decoupling with an RF field strength of 10 kHz. The *t*_1_ period was incremented up to 5.1 ms with a *t*_1_ increment of 0.15 ms. The ^1^H polarization was transferred to the ^13^C spins by adiabatic tangential double-quantum cross polarization (DQ-CP), which was identical to the first CP scheme in Fig B in S1 File. The contact time for CP was 1.5ms. During the acquisition (*t*_2_) period of 10.2 ms, SPINAL-64 ^1^H decoupling and WALTZ-16 ^2^H decoupling were applied with RF strengths of 10 kHz and 5 kHz, respectively. The *t*_2_ dwell time was 5 μs. The phase cycles for the pulse sequence were as follows: *ϕ*_1_ = y, -y; *ϕ*_2_ = x, x, -x, -x; *ϕ*_3_ = y; *ϕ*_4_ = x, -x, -x, x. The phase *ϕ*_1_ and the receiver phase were incremented along the *t*_1_ points using the States-TPPI data collection mode. **Fig. D, A pulse sequence used for** ^**1**^ **H-detected 3D** ^**13**^ **C/** ^**13**^ **C/** ^**1**^ **H correlation spectroscopy in Fig 3**. ^13^C spin polarization was prepared by adiabatic double-quantum cross polarization (DQ-CP) using the same parameters as discussed in Fig B in S1 File. During the *t*_1_ period, SPINAL-64 ^1^H decoupling and WALTZ-16 ^2^H decoupling were applied with RF field strengths of 10 kHz and 5 kHz, respectively. After the *t*_1_ period, a transverse component of the ^13^C polarization was stored along the z-axis and the unnecessary component in the transverse plane is dephased during a z-filter period τ of 2 ms. Then, ^13^C polarization transfer was achieved by ^13^C-^13^C dipolar couplings using the fpRFDR sequence without ^1^H rf irradiation. A π-pulse train with the XY-16 phase cycle was rotor-synchronously applied to the ^13^C channel so that a π-pulse was applied at the center of every rotor cycle. The π-pulse width in the fpRFDR mixing was 6.6 μs, and *n* = 96. After a z-filter and excitation by a π/2-pulse, ^13^C signals were recorded during the *t*_2_ period under SPINAL-64 ^1^H decoupling and WALTZ-16 ^2^H decoupling, as mentioned above for the *t*_1_ period. Then, a transverse component of the ^13^C polarization was transferred back to ^1^H spins by an adiabatic DQ-CP scheme before the acquisition of ^1^H signals in the *t*_3_ period. The ^1^H RF field strength was swept from 26.4 kHz to 66.0 kHz with the average rf field at 46.2 kHz (~3ν_R_/5) while the ^13^C RF field amplitude was set kept constant at 32.0 kHz (~2ν_R_/5). The contact time of the second CP period was 0.5 ms. The *t*_1_ and *t*_2_ periods were both incremented up to 2.4 ms with an increment of 75 μs. The *t*_3_ acquisition time was 10.2 ms with 5 μs dwell time. The phase cycles for the pulse sequence were as follows: *ϕ*_1_ = y; *ϕ*_2_ = x; *ϕ*_3_ = x, x, -x, -x; *ϕ*_4_ = y, y, y, y, -y, -y, -y, -y; *ϕ*_5_ = y; *ϕ*_6_ = x, -x; *ϕ*_7_ = x; *ϕ*_8_ = x, -x, -x, x, -x, x, x, -x. The phases *ϕ*_3_ and *ϕ*_5_ and the receiver phase were incremented along the *t*_1_ and *t*_2_ points using the States-TPPI data collection mode. **Fig. E, a)** ^**1**^ **H-detected** ^**13**^ **C/** ^**1**^ **H 2D correlation and b)** ^**13**^ **C-detected** ^**13**^ **C/** ^**1**^ **H 2D correlation, and c) 1D** ^**13**^ **C CP-MAS spectra of SAIL-Ile respectively**. 1D slices at various ^1^H chemical shifts (indicated in the fig.) from the ^1^H and ^13^C detected 2D ^13^C/^1^H correlation spectra are compared. The 1D slices and 1D spectrum in (c) are scaled so that all the 1D spectra show a common noise level for sensitivity comparisons. The experimental time was 5 min each. The pulse sequences used for (a) ^13^C-detected and (b) ^1^H-detected 2D ^1^H/^13^C chemical-shift correlation experiments are shown in Fig C and Fig B in S1 File, respectively. The CP and decoupling conditions for these experiments were similar to those for the data for the SAIL Ile labeled ubiquitin sample in Fig 2. The ^13^C detection/evolution periods was 10 ms, while ^1^H detection/evolution periods was 6.5 ms for a) and b). These periods were matched to the inverse of the average line widths of ^13^C and ^1^H. Although ^1^H T_1_ value for this sample was ~3 s, the recycle delay was set to 0.3 s as sufficient signal-to-noise ratios can be obtained for all of (a-c). All the spectra in Fig. E were processed with 45̊- and 60̊-shifted sinebell functions on the ^1^H and ^13^C dimensions respectively without linear prediction. **Fig. F, A comparison of 1D** ^**13**^ **C MAS spectra of SAIL-Ile by a) π/2-pulse direct excitation and b) cross-polarization (CP) from** ^**1**^ **H spins**. The pulse sequence for cross polarization experiments used in (b) was the same as showed in Fig C in S1 File except that the *t*_1_ value was set to 0.1 μs and *t*_2_ was used as an acquisition period. The cross polarization transfer was optimized for protonated carbons for ^1^H-detected experiments. The ^1^H RF field strength was swept from 70.0 kHz to 28.0 kHz with the average rf field set at 49.0 kHz (~5ν_R_/8) while the ^13^C RF field strength was kept constant at 30.0 kHz (~3ν_R_/8). The contact time of CP was 1.5 ms. The ^13^C detection periods was 3.1 ms for both (a) and (b). Recycle delays were set to 6000 s and 20 s for (a) and (b), respectively. The long delays were employed to ensure that the signals were fully recovered. No window functions were applied to the spectra. The CP-transfer efficiency for C_α_, C_β_, C_γ1_, C_γ2_ and C_δ_ were 55%, 60%, 68%, 45% and 40%, respectively. The values were obtained by dividing the ratio of the integral peak intensity in (b) to that of the corresponding peak in (a) by γ_H_/γ_C_, where γ_H_ and γ_C_ are the gyromagnetic ratios of ^1^H and ^13^C, respectively. **Table A, The comparison of S/N for** ^**1**^ **H-detected 2D** ^**13**^ **C/** ^**1**^ **H correlation**, ^**13**^ **C-detected** ^**13**^ **C/** ^**1**^ **H correlation, and** ^**13**^ **C 1D CPMAS experiments of the SAIL-Ile labeled ubiquitin sample. Table B, The comparison of signal-to-noise ratios (S/N) for** ^**1**^ **H-detected 2D** ^**13**^ **C/** ^**1**^ **H correlation**, ^**13**^ **C-detected** ^**13**^ **C/** ^**1**^ **H correlation, and** ^**13**^ **C 1D CPMAS experiments of SAIL Ile sample. Table C, Preliminary** ^**1**^ **H and** ^**13**^ **C signal assignments of SAIL-Ile labeled ubiquitin**.
(PDF)
