# Supplementary material for: Nano-Mole Scale Side-Chain Signal Assignment by 1H-Detected Protein Solid-State NMR by Ultra-Fast Magic-Angle Spinning and Stereo-Array Isotope Labeling
Source: PLoS One. 2015 Apr 9;10(4):e0122714. doi: 10.1371/journal.pone.0122714 (PMC4391754; doi:10.1371/journal.pone.0122714)
Supplement: S1 File — The 1H NMR spectra were obtained using WALTZ-16 13C decoupling with an RF field strength of 10 kHz. All the spectra were obtained with 2 scans with a pulse delay of 15 s, and the data were processed without any window function. The 1H line widths for Hα, Hβ, Hγ OH/NH are 0.22 ppm, 0.22 ppm, 0.24 ppm, 0.20 ppm, respectively. Fig. B, A pulse sequence for 1 H-detected 2D 1 H/ 13 C chemical-shift correlation spectroscopy used for Fig 2a. In this sequence, 13C spin polarization was prepared with adiabatic double-quantum cross polarization (DQ-CP) using an amplitude-modulated shaped pulse with a downward tangential ramp for the 1H channel and a rectangular pulse for the 13C channel. The 1H RF field strength was swept from 66.0 kHz to 26.4 kHz with the average rf field set at 46.2 kHz (~3νR/5) while the 13C RF field amplitude was kept constant at 32.0 kHz (~2νR/5). The contact time of the first CP was 1.5ms. During the t 1 period, SPINAL-64 1H decoupling[38] and WALTZ-16 2H decoupling were applied with RF field strengths of 10 kHz and 5 kHz, respectively. The t 1 period was incremented up to 5.1 ms with an increment of 37 μs. After the t 1 period, a pair of π/2-pulses were applied as a Z-filter in order to select the real or imaginary component of the 13C polarization, which was transferred back to 1H spins with the second adiabatic CP using a reversed upward tangential ramp for the 1H channel and the same rectangular pulse for the 13C channel. The contact time of the second CP was 1.5 ms. During the acquisition (t 2) period of 10.2 ms, 1H signals were acquired with dwell times of 5 μs under 13C decoupling using WALTZ-16 sequence[41] with an RF field strength of 10 kHz. The phase cycles for the pulse sequence were as follows: ϕ 1 = y; ϕ 2 = x; ϕ 3 = x, x, -x, -x; ϕ 4 = y, y, y, y, -y, -y, -y, -y; ϕ 5 = x; ϕ 6 = y, -y; ϕ 7 = y; ϕ 8 = x, -x, -x, x, -x, x, x, -x. The phase ϕ 3 and the receiver phase were incremented along the t 1 points using the States-TPPI data collection m [file pone.0122714.s001.pdf]

Supporting information for "Nano-mole Scale Side-chain Signal Assignment by ^{1}H-Detected Protein Solid-state NMR by Ultra-Fast Magic-Angle Spinning and Stereo-Array Isotope Labeling "

by Songlin Wang, Sudhakar Parthasarathy, Yusuke Nishiyama, Yuki Endo, Takahiro Nemoto, Kazuo Yamauchi, Tetsuo Asakura, Mitsuhiro Takeda, Tsutomu Terauchi, Masatsune Kainosho, and Yoshitaka Ishii

Supplementary Figs.

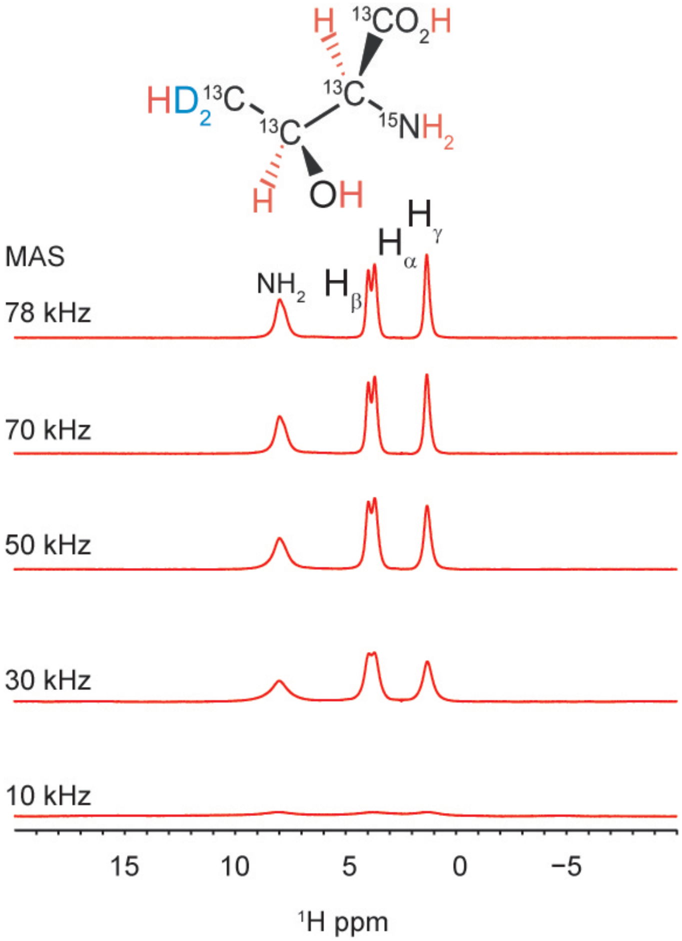

Fig. A. Spinning speed dependence of ^{1}H MAS SSNMR of SAIL-threonine (SAIL-Thr) with its chemical structure and labeling scheme at the top. The ^{1}H NMR spectra were obtained using WALTZ-16 ^{13}C decoupling with an RF field strength of 10 kHz. All the spectra were obtained with 2 scans with a pulse delay of 15 s, and the data were processed without any window function. The ^{1}H line widths for H_{α}, H_{β}, H_{γ} OH/NH are 0.22 ppm, 0.22 ppm, 0.24 ppm, 0.20 ppm, respectively.

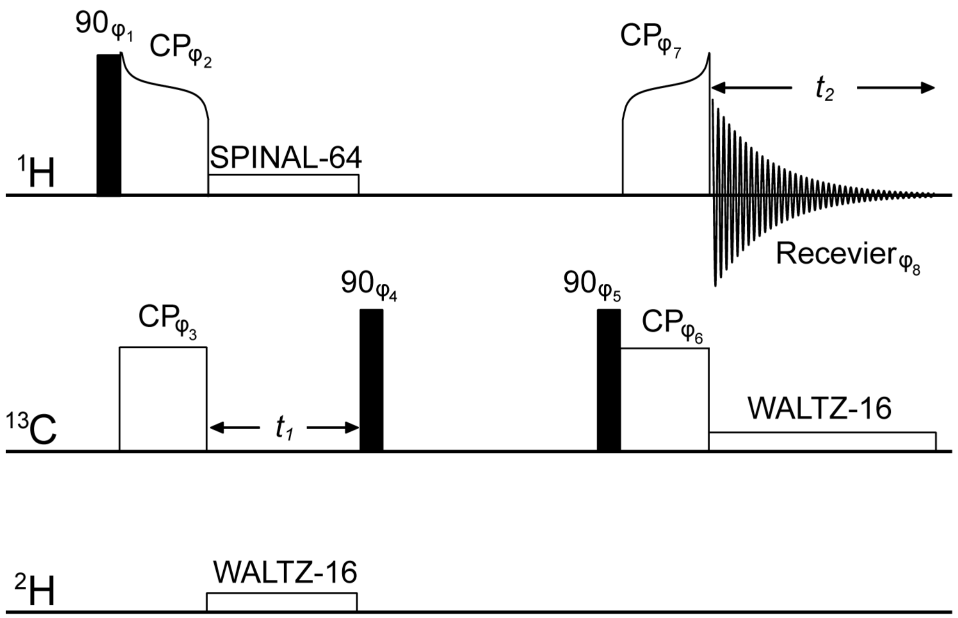

Fig. B. A pulse sequence for ^{1}H-detected 2D ^{1}H/^{13}C chemical-shift correlation spectroscopy used for Fig. 2a. In this sequence, ^{13}C spin polarization was prepared with adiabatic double-quantum cross polarization (DQ-CP) using an amplitude-modulated shaped pulse with a downward tangential ramp for the ^{1}H channel and a rectangular pulse for the ^{13}C channel. The ^{1}H RF field strength was swept from 66.0 kHz to 26.4 kHz with the average rf field set at 46.2 kHz (~$3\nu_R/5$) while the ^{13}C RF field amplitude was kept constant at 32.0 kHz (~$2\nu_R/5$). The contact time of the first CP was 1.5ms. During the t_1 period, SPINAL-64 ^{1}H decoupling[1] and WALTZ-16 ^{2}H decoupling were applied with RF field strengths of 10 kHz and 5 kHz, respectively. The t_1 period was incremented up to 5.1 ms with an increment of 37 μs. After the t_1 period, a pair of π/2-pulses were applied as a Z-filter in order to select the real or imaginary component of the ^{13}C polarization, which was transferred back to ^{1}H spins with the second adiabatic CP using a reversed upward tangential ramp for the ^{1}H channel and the same rectangular pulse for the ^{13}C channel. The contact time of the second CP was 1.5 ms. During the acquisition (t_2) period of 10.2 ms, ^{1}H signals were acquired with dwell times of 5 μs under ^{13}C decoupling using WALTZ-16 sequence[2] with an RF field strength of 10 kHz. The phase cycles for the pulse sequence were as follows: $\phi_1 =$ y; $\phi_2 =$ x; $\phi_3 =$ x, x, -x, -x; $\phi_4 =$ y, y, y, y, -y, -y, -y, -y; $\phi_5 =$ x; $\phi_6 =$ y, -y; $\phi_7 =$ y; ϕ_8 = x, -x, -x, x, -x, x, x, -x. The phase ϕ_3 and the receiver phase were incremented along the t_1 points using the States-TPPI data collection mode.

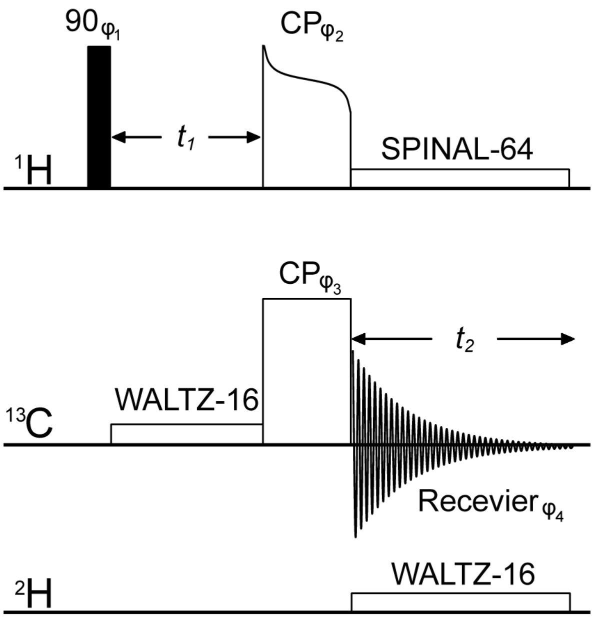

Fig. C. A pulse sequence used for ^{13}C-detected 2D ^{1}H/^{13}C chemical-shift correlation spectroscopy in Fig. 2b. After excitation by a π/2-pulse, ^{1}H spin polarization evolved under ^{1}H chemical-shift interactions during the t_1 period under WALTZ-16 ^{13}C decoupling with an RF field strength of 10 kHz. The t_1 period was incremented up to 5.1 ms with a t_1 increment of 0.15 ms. The ^{1}H polarization was transferred to the ^{13}C spins by adiabatic tangential double-quantum cross polarization (DQ-CP), which was identical to the first CP scheme in S2 Fig. The contact time for CP was 1.5ms. During the acquisition (t_2) period of 10.2 ms, SPINAL-64 ^{1}H decoupling and WALTZ-16 ^{2}H decoupling were applied with RF strengths of 10 kHz and 5 kHz, respectively. The t_2 dwell time was 5 μs. The phase cycles for the pulse sequence were as follows: ϕ_1 = y, -y; ϕ_2 = x, x, -x, -x; ϕ_3 = y; ϕ_4 = x, -x, -x, x. The phase ϕ_1 and the receiver phase were incremented along the t_1 points using the States-TPPI data collection mode.

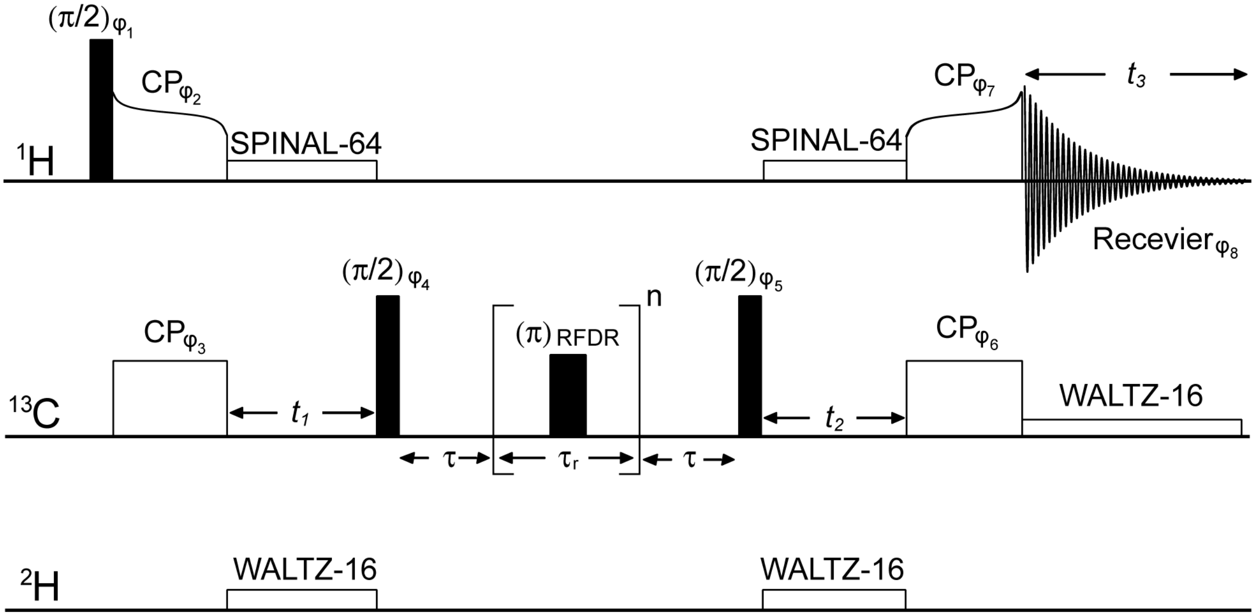

Fig. D. A pulse sequence used for ^{1}H-detected 3D ^{13}C/^{13}C/^{1}H correlation spectroscopy in Fig. 3. ^{13}C spin polarization was prepared by adiabatic double-quantum cross polarization (DQ-CP) using the same parameters as discussed in S2 Fig. During the t_1 period, SPINAL-64 ^{1}H decoupling and WALTZ-16 ^{2}H decoupling were applied with RF field strengths of 10 kHz and 5 kHz, respectively. After the t_1 period, a transverse component of the ^{13}C polarization was stored along the z-axis and the unnecessary component in the transverse plane is dephased during a z-filter period τ of 2 ms. Then, ^{13}C polarization transfer was achieved by ^{13}C-^{13}C dipolar couplings using the fpRFDR sequence without ^{1}H rf irradiation. A π-pulse train with the XY-16 phase cycle was rotor-synchronously applied to the ^{13}C channel so that a π-pulse was applied at the center of every rotor cycle. The π-pulse width in the fpRFDR mixing was 6.6 μs, and n = 96. After a z-filter and excitation by a $\pi/2$-pulse, ^{13}C signals were recorded during the t_2 period under SPINAL-64 ^{1}H decoupling and WALTZ-16 ^{2}H decoupling, as mentioned above for the t_1 period. Then, a transverse component of the ^{13}C polarization was transferred back to ^{1}H spins by an adiabatic DQ-CP scheme before the acquisition of ^{1}H signals in the t_3 period. The ^{1}H RF field strength was swept from 26.4 kHz to 66.0 kHz with the average rf field at 46.2 kHz (~$3\nu_R/5$) while the ^{13}C RF field amplitude was set kept constant at 32.0 kHz (~$2\nu_R/5$). The contact time of the second CP period was 0.5 ms. The t_1 and t_2 periods were both incremented up to 2.4 ms with an increment of 75 μs. The t_3 acquisition time was 10.2 ms with 5 μs dwell time. The phase cycles for the pulse sequence were as follows: ϕ_1 = y; ϕ_2 = x; ϕ_3 = x, x, -x, -x; ϕ_4 = y, y, y, y, -y, -y, -y, -y; ϕ_5 = y; ϕ_6 = x, -x; ϕ_7 = x; ϕ_8 = x, -x, -x, x, -x, x, x, -x. The phases ϕ_3 and ϕ_5 and the receiver phase were incremented along the t_1 and t_2 points using the States-TPPI data collection mode.

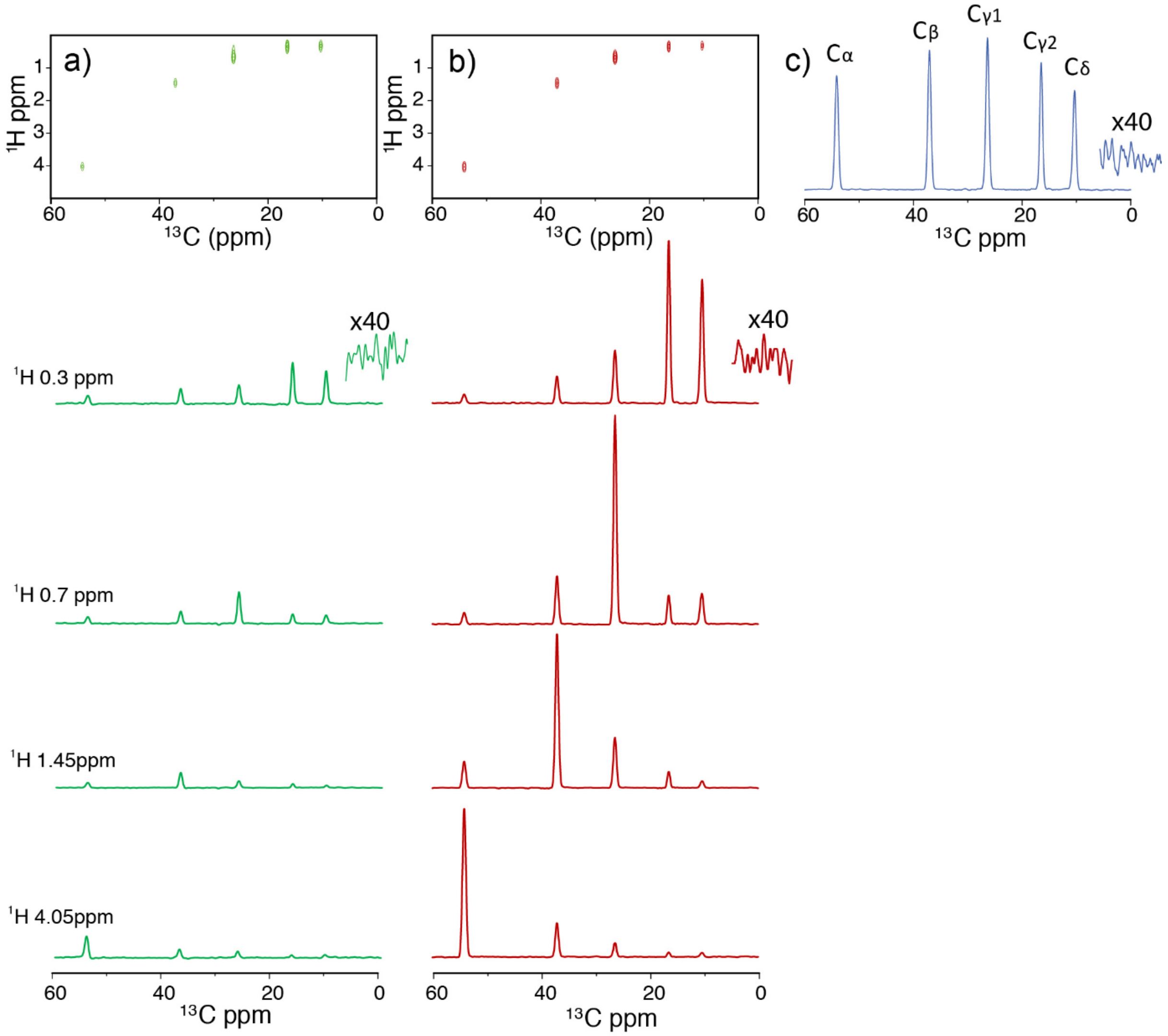

Fig. E. a) ^{13}C-detected ^{13}C/^{1}H 2D correlation and b) ^{1}H-detected ^{13}C/^{1}H 2D correlation, and c) 1D ^{13}C CP-MAS spectra of SAIL-Ile respectively. 1D slices at various ^{1}H chemical shifts (indicated in the fig.) from the ^{1}H and ^{13}C detected 2D ^{13}C/^{1}H correlation spectra are compared. The 1D slices and 1D spectrum in (c) are scaled so that all the 1D spectra show a common noise level for sensitivity comparisons. The experimental time was 5 min each. The pulse sequences used for (a) ^{13}C-detected and (b) ^{1}H-detected 2D ^{1}H/^{13}C chemical-shift correlation experiments are shown in S3 Fig. and S2 Fig., respectively. The CP and decoupling conditions for these experiments were similar to those for the data for the SAIL Ile labeled ubiquitin sample in Fig. 2. The ^{13}C detection/evolution periods was 10 ms, while ^{1}H detection/evolution periods was 6.5 ms for a) and b). These periods were matched to the inverse of the average line widths of ^{13}C and ^{1}H. Although ^{1}H T_1 value for this sample was ~3 s, the recycle delay was set to 0.3 s as sufficient signal-to-noise ratios can be obtained for all of (a-c). All the spectra in S5 Fig. were processed with 45°- and 60°-shifted sinebell functions on the ^{1}H and ^{13}C dimensions respectively without linear prediction.

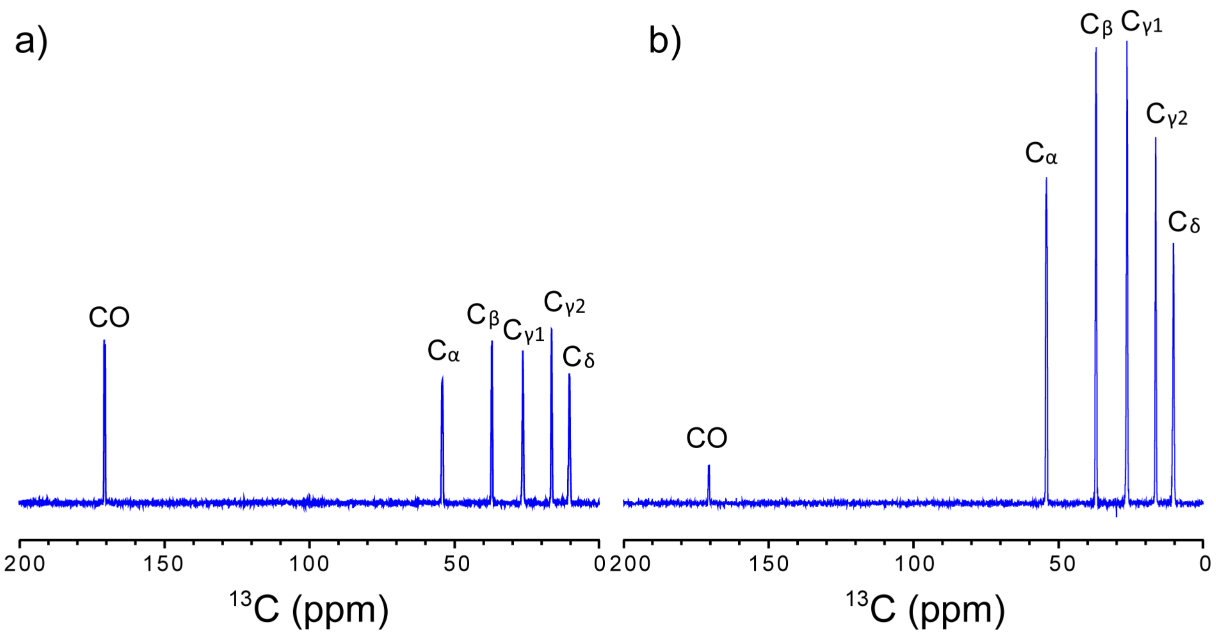

Fig. F. A comparison of 1D ^{13}C MAS spectra of SAIL-Ile by a) π/2-pulse direct excitation and b) cross-polarization (CP) from ^{1}H spins. The pulse sequence for cross polarization experiments used in (b) was the same as showed in S3 Fig. except that the t_1 value was set to 0.1 μs and t_2 was used as an acquisition period. The cross polarization transfer was optimized for protonated carbons for ^{1}H-detected experiments. The ^{1}H RF field strength was swept from 70.0 kHz to 28.0 kHz with the average rf field set at 49.0 kHz (~5ν_R/8) while the ^{13}C RF field strength was kept constant at 30.0 kHz (~3ν_R/8). The contact time of CP was 1.5 ms. The ^{13}C detection periods was 3.1 ms for both (a) and (b). Recycle delays were set to 6000 s and 20 s for (a) and (b), respectively. The long delays were employed to ensure that the signals were fully recovered. No window functions were applied to the spectra. The CP-transfer efficiency for C_α, C_β, $C_{\gamma1}$, $C_{\gamma2}$ and C_δ were 55%, 60%, 68%, 45% and 40%, respectively. The values were obtained by dividing the ratio of the integral peak intensity in (b) to that of the corresponding peak in (a) by γ_H/γ_C, where γ_H and γ_C are the gyromagnetic ratios of ^{1}H and ^{13}C, respectively.

Preparation of SAIL-Thr microcrystals

SAIL-Thr powder was recrystallized by dissolving 3.7 mg of the amino acid in 35 μL D_2O followed by the addition of 55 μL d_4-methanol. Precipitate was observed after the addition of d_4-methanol and it was allowed to stand at room temperature for slow evaporation. The dry crystals were recovered after 2 weeks, and the sample was used for the SSNMR measurements.

Preparation of SAIL-Ile labeled Ubq

E. coli BL21 (DE3) cells transformed with a pET3 vector (Novagen, Madison, WI) encoding chlorella Ubq were grown in 500 mL of D_2O/M9 medium supplemented with 2.5 mg of SAIL-Ile (SAIL Technologies, Sagamihara, Japan) at 37 ºC up to an OD_{600} of 0.6. At this time, 5.0 mg of SAIL Ile was additionally supplemented and then Ubq expression was induced by adding isopropyl β-D-1-thiogalactopyranoside. After 8 h induction, the cells were collected and then the produced protein was purified by ion-exchange chromatography on a DE52 column, a HiTrap SP column and then a gel filtration chromatography on a Superdex 200 column (All column materials from GE Healthcare, Uppsala, Sweden). The yield of SAIL-Ile labeled Ubq was ~14 mg, and the labeling efficiency of ~90 % was confirmed by mass spectroscopy.

Estimation of the α-factors in eq. [1] for different window functions

Following ref. [3], the frequency-domain signal-to-noise ratio (S/N) per root of the experimental time in an one-dimensional (1D) direct detection of an X-nuclei with CP is expressed as

$$(S/N)_{\mathrm{DD\_1D}} = f_{\mathrm{HX}}(\gamma_{\mathrm{X}} B_0)^{3/2}\gamma_{\mathrm{H}} \frac{\langle s_{\mathrm{X}}(t) w_{\mathrm{X}}(t)\rangle}{\langle w_{\mathrm{X}}^2(t)\rangle^{1/2}} (t^{\max})^{1/2} Q_{\mathrm{X}}^{1/2} C_{\mathrm{X}}, \qquad \text{[S1]}$$

where B_0 is a static magnetic field, γ_A is a gyromagnetic ratio for nuclei A, f_{HX} is the efficiency of CP transfer from ^{1}H to X ($0 \leq f_{HX} \leq 1$), w_X(t) is an apodization window function, t_{max} is the length of the acquired time-domain signal for the X nuclei, and $\langle z(t) \rangle$ denotes the time average of z over the interval [0, t_{max}]. The normalized 1D time-domain NMR signal of the X nuclei is assumed to be of the form $s_X(t)\exp(-i\omega_X t)$, where $s_X(t)$ is a non-negative envelope function with

$s_X(0) = 1$. Here, Q_X is a quality factor of the sample coil for X detection; C_X is a constant that includes properties such as temperature, coil geometry, filling factor, receiver noise figure, spin density, and repetition rate for signal averaging. The frequency-domain S/N per root of the experimental time in a two-dimensional (2D) ^{1}H indirect detection of X nuclei (*i.e.* ^{1}H-detected H/X HETCOR) is expressed as

$$(S/N)_{\mathrm{ID\_2D}} = \frac{1}{\sqrt{2}} f_{\mathrm{XH}} f_{\mathrm{HX}} (\gamma_{\mathrm{H}} B_0)^{3/2} \gamma_{\mathrm{H}} \frac{\langle s_{\mathrm{ID}}(t_1,t_2) w_{\mathrm{ID}}(t_1,t_2)\rangle}{\langle w_{\mathrm{ID}}^2(t_1,t_2)\rangle^{1/2}} (t_{2\mathrm{H}}^{\max})^{1/2} Q_{\mathrm{H}}^{1/2} C_{\mathrm{H}}, \qquad [S2]$$

where f_{XH} is the efficiency of CP transfer from X to ^{1}H ($0 \le f_{XH} \le 1$), $s_{ID}(t_1, t_2)$ is a normalized envelope function of the 2D time-domain H/X correlation signal for the indirect detection with $s_{ID}(0, 0) = 1$. $w_{ID}(t_1, t_2)$ is a window function for the 2D data, $t_{kA}^{\max}$ (k = 1, 2) is the maximum t_k value for recording signals of nuclei A (A = H or X), and $\langle z(t_1, t_2)\rangle$ denotes the time average of z over the intervals [0, $t_{1X}^{\max}$] and [0, $t_{2H}^{\max}$]. Here, Q_H is the quality factor of the sample coil for the ^{1}H channel and C_H is a constant analogous to C_X. If the direct and indirect detection experiments are performed at the same repetition rate and at the same temperature using a probe with a single double-tuned sample coil for X and ^{1}H nuclei, we can assume $C_H \sim C_X$. The factor $1/\sqrt{2}$ in eq. [S2] is attributed to quadrature detection in the t_1 period. When the 2D signal can be separated as $s_{ID}(t_1, t_2) = s_X(t_1)s_H(t_2)$ and $w(t_1, t_2) = w_X(t_1)w_H(t_2)$, eq. [S2] can be rewritten as

$$(S/N)_{\mathrm{ID\_2D}} = \frac{1}{\sqrt{2}} f_{\mathrm{XH}} f_{\mathrm{HX}} (\gamma_{\mathrm{H}} B_0)^{3/2} \gamma_{\mathrm{H}} \frac{\langle s_{\mathrm{X}}(t_1) w_{\mathrm{X}}(t_1)\rangle}{\langle w_{\mathrm{X}}^2(t_1)\rangle^{1/2}} \frac{\langle s_{\mathrm{H}}(t_2) w_{\mathrm{H}}(t_2)\rangle}{\langle w_{\mathrm{H}}^2(t_2)\rangle^{1/2}} (t_{2\mathrm{H}}^{\max})^{1/2} Q_{\mathrm{H}}^{1/2} C_{\mathrm{H}}. \qquad [S3]$$

Similarly, the frequency-domain S/N per root of the experimental time in a 2D ^{1}H/X HETCOR via direct X detection is expressed as

$$(S/N)_{\mathrm{DD\_2D}} = \frac{1}{\sqrt{2}} f_{\mathrm{HX}} (\gamma_{\mathrm{X}} B_0)^{3/2} \gamma_{\mathrm{H}} \frac{\langle s_{\mathrm{DD}}(t_1,t_2) w_{\mathrm{DD}}(t_1,t_2)\rangle}{\langle w_{\mathrm{DD}}^2(t_1,t_2)\rangle^{1/2}} (t_{2\mathrm{X}}^{\max})^{1/2} Q_{\mathrm{X}}^{1/2} C_{\mathrm{X}}$$

$$= \frac{1}{\sqrt{2}} f_{\mathrm{HX}} (\gamma_{\mathrm{X}} B_0)^{3/2} \gamma_{\mathrm{H}} \frac{\langle s_{\mathrm{H}}(t_1) w_{\mathrm{H}}(t_1)\rangle}{\langle w_{\mathrm{H}}^2(t_1)\rangle^{1/2}} \frac{\langle s_{\mathrm{X}}(t_2) w_{\mathrm{X}}(t_2)\rangle}{\langle w_{\mathrm{X}}^2(t_2)\rangle^{1/2}} (t_{2\mathrm{X}}^{\max})^{1/2} Q_{\mathrm{X}}^{1/2} C_{\mathrm{X}}, \qquad [S4]$$

where $s_{DD}(t_1, t_2)$ is the normalized envelope function of the 2D time-domain signal for the direct detection, $s_{DD}(0, 0) = 1$, and $w_{DD}(t_1, t_2)$ is a window function for the 2D data. Here, the time average for $<z(t_1, t_2)>$ is over the intervals $[0, t_{1H}^{max}]$ and $[0, t_{2X}^{max}]$.

First, we compare the sensitivity of 2D ^{1}H indirect detection with that of 1D X direct detection. In case that $C_X \sim C_H$ and $t^{max} = t_{1X}^{max}$, the sensitivity enhancement factor ξ_{1D} by 2D ^{1}H indirect detection over 1D X-detection is given by

$$\xi_{1D} = \frac{(S/N)_{ID\_2D}}{(S/N)_{DD\_1D}} = \frac{f_{XH}}{\sqrt{2}}\left(\frac{\gamma_H}{\gamma_X}\right)^{3/2} \frac{\langle s_H(t_2) w_H(t_2)\rangle}{\langle w_H(t_2)\rangle^{1/2}} \left(\frac{t_{2H}^{max}}{t_{1X}^{max}}\right)^{1/2} \left(\frac{Q_H}{Q_X}\right)^{1/2}. \qquad [S5]$$

Assuming that t_{2H}^{max} and t_{1X}^{max} are set proportionally to the inverses of the ^{1}H line width W_H and the X line width W_X, respectively, $t_{2H}^{max} / t_{1X}^{max} = W_X/W_H$, where W_A represents a general spectral line width (in Hz) defined by

$$W_A = \frac{1}{2\pi\tau^{max}\langle s_A(\tau) w_A(\tau)\rangle}, \qquad [S6]$$

where τ^{max} denotes the maximum length of τ. For a Lorentzian line shape, W_A is reduced to the conventional half-width-at-half-height (HWHH) width after applying a Lorentzian window function assuming that $s_A(\tau^{max})\, w_A(\tau^{max}) \sim 0$. Then,

$$\xi_{1D} = \frac{(S/N)_{ID\_2D}}{(S/N)_{DD\_1D}} = \frac{f_{XH}}{\sqrt{2}}\left(\frac{\gamma_H}{\gamma_X}\right)^{3/2} \frac{\langle s_H(t_2) w_H(t_2)\rangle}{\langle w_H(t_2)\rangle^{1/2}} \left(\frac{W_X}{W_H}\right)^{1/2} \left(\frac{Q_H}{Q_X}\right)^{1/2}. \qquad [S7]$$

Thus, the α factor in eq. [1] depends on $\langle s_H(t_2) w_H(t_2)\rangle / \langle w_H(t_2)\rangle^{1/2}$. Now, we evaluate sensitivity enhancement factor by 2D indirect detection over 2D direct detection ξ_{2D} as

$$\xi_{2D} = \frac{(S/N)_{ID\_2D}}{(S/N)_{DD\_2D}} = f_{XH}\left(\frac{\gamma_H}{\gamma_X}\right)^{3/2} \left(\frac{t_{2H}^{max}}{t_{2X}^{max}}\right)^{1/2} \left(\frac{Q_H}{Q_X}\right)^{1/2}. \qquad [S8]$$

Hence, $\alpha = 1$ in eq. [1] in this case when $t_{2H}^{max} / t_{2X}^{max} = W_X/W_H$. In case that $t_{2X}^{max} = t_{1X}^{max}$, the ratio of ξ_{2D} to ξ_{1D} (ζ) is given by

$$\zeta \equiv \frac{\xi_{1D}}{\xi_{2D}} = \frac{1}{\sqrt{2}} \frac{\langle s_{\mathrm{H}}(t_2) w_{\mathrm{H}}(t_2) \rangle}{\langle w_{\mathrm{H}}^2(t_2) \rangle^{1/2}} . \qquad \text{[S9]}$$

It is noteworthy that ζ = (S/N)$_{DD\_2D}$/(S/N)$_{DD\_1D}$. In general, the ratio ζ reflects the ^{1}H signal envelope and window functions as well as a factor of $1/\sqrt{2}$ due to quadrature detection. When a matched window function of $w_{\mathrm{H}}(t_2) = s_{\mathrm{H}}(t_2)$ is used,

$$\zeta = \frac{1}{\sqrt{2}} \langle s_{\mathrm{H}}^2(t_2) \rangle^{1/2} . \qquad \text{[S10]}$$

With a matched window function, $t_{2H}^{\max}$ is typically selected to be ~1/(2W_{H}) for a Lorentzian line shape, where 2W_{H} represents a full-width-at-half-height (FWHH) width *after broadening*. From eq. [S6], $W_{\mathrm{H}} = 1/\{2\pi t_{2\mathrm{H}}^{\max} \langle s_{\mathrm{H}}(t)^2 \rangle\}$ and we obtain $\langle s_{\mathrm{H}}(t)^2 \rangle = 1/\pi$. Thus, $\zeta = 1/\sqrt{2\pi}$ ~1/2.5.

In biomolecular SSNMR, particularly for uniformly or heavily ^{13}C-labeled samples, no or minimal window functions are often applied to gain sufficient spectral resolution. When no window function is applied (i.e. $w_{\mathrm{H}}(t)$ =1), $t_{2H}^{\max}$ is typically selected to be ~1/(2W_{H}) or the inverse of the FWHH width for a Lorentzian line shape. In this case, we obtain $\zeta = 1/(\sqrt{2}\pi)$ (or ~1/4.4) from eq. [S6] and eq. [S9]. Therefore, it becomes more difficult to enhance sensitivity by ^{1}H indirect detection over 1D ^{13}C CPMAS when no window functions are applied on ^{1}H for higher resolution. A similar situation using resolution enhancement window functions is typical in protein SSNMR, in which ζ is often in a range of 1/5 to 1/4.

Table A. The comparison of S/N for ^{1}H-detected 2D $^{13}C/^{1}H$ correlation, ^{13}C-detected $^{13}C/^{1}H$ correlation, and ^{13}C 1D CPMAS experiments of the SAIL-Ile labeled ubiquitin sample.

Peak position (ppm)		S/N (Relative S/N with respect to that of ^{1}H detected 2D)[d)]		
^{13}C shift[a)]	^{1}H shift[a)]	^{1}H-detect 2D	^{13}C-detect 2D[b)]	^{13}C-detect 1D[c)]
7.8	0.43	12.1	1.6 (1/7.6)	2.4 (1/5.0)
13.3	0.67	11.3	2.1 (1/5.4)	
13.5	0.42	17.8	2.5 (1/7.1)	
13.9	0.60	14.5	1.5 (1/9.7)	
14.8	0.86	11.5	1.6 (1/7.2)	
15.9	0.55	18.6	2.6 (1/7.2)	
16.0	0.32	16.2	1.8 (1/9.0)	
17.0	0.68	26.4	3.1 (1/8.5)	
17.1	0.49	15.8	2.5 (1/6.3)	
24.5	0.69	10.0	1.5 (1/6.7)	4.8 (1/2.1)
26.3	1.83	12.0	*	
26.6	0.89	5.5	*	
27.6	0.34	8.3	*	
27.7	0.84	11.5	*	
30.5	0.55	17.8	2.3 (1/7.7)	7.1 (1/2.5)
36.0	0.55	7.2	*	
36.1	2.47	8.4	*	
36.6	1.38	10.4	*	
42.4	1.49	8.7	*	

a) The peak positions were obtained from the ^{1}H-detected 2D $^{1}H/^{13}C$ correlation spectrum. The chemical shifts were referenced to DSS.

b) The S/N values were estimated from the sum spectrum of two separate ^{13}C-detect 2D spectra collected with 5 min each, and the S/N values were divided by $\sqrt{2}$. The S/N values are not reported for the peaks noted by * as these peaks were under the detection limit.

c) The S/N values were estimated from the sum spectrum of four separate ^{13}C 1D CPMAS spectra collected with 5 min each, and the values were divided by 2. Only 3 peaks were analyzed as other peaks were overlapping in the 1D spectrum.

d) The S/N value here is defined by the peak intensity (S) divided by twice the root-mean-square noise level σ_N ($S/2\sigma_N$). The relative S/N of $1/p$ means that ^{1}H-detected scheme enhances S/N by a factor of p.

Table B. The comparison of signal-to-noise ratios (S/N) for ^{1}H-detected 2D ^{13}C/^{1}H correlation, ^{13}C-detected ^{13}C/^{1}H correlation, and ^{13}C 1D CPMAS experiments of SAIL Ile sample.

	Cα	Cβ	Cγ1	Cγ2	Cδ
^{1}H detect 2D$^{b)}$	317.7	520.3	653.0	348.9	271.9
^{13}C detect 2D$^{a, b)}$	46.7<br>(1/6.80)	51.6<br>(1/10.08)	98.4<br>(1/6.64)	89.4<br>(1/3.90)	70.2<br>(1/3.87)
^{13}C detect 1D$^{a. b)}$	248.7<br>(1/1.28)	301.4<br>(1/1.73)	329.8<br>(1/1.98)	272.9<br>(1/1.28)	217.2<br>(1/1.25)

a) The values in the parentheses denote relative S/N with respect to the corresponding S/N ratio for the ^{1}H detected 2D correlation experiment.

b) The data are shown in S5 Fig. The S/N value here is defined by the peak intensity divided by twice the root-mean-square noise level σ_N ($S/2\sigma_N$).

Table C. Preliminary signal assignments of SAIL-Ile labeled ubiquitin.

Residue$^{a)}$	Chemical Shifts (ppm)								
	$^{13}C_\alpha$	$^{13}C_\beta$	$^{13}C_{\gamma 1}$	$^{13}C_{\gamma 2}$	$^{13}C_\delta$	$^{1}H_\beta$	$^{1}H_{\gamma 1}$	$^{1}H_{\gamma 2}$	$^{1}H_\delta$
Ile3	59.2	41.8	24.5	17.1	13.3	1.57	0.65	0.40	0.37
Ile13	59.8	40.9	27.4	17.2	14.1	1.73	-$^{b)}$	0.50	0.47
Ile23	61.5	34.4	26.3	17.2	7.8	2.09	1.77	0.63	0.41
Ile30	66.0	36.1	30.4	15.9	14.7	2.42	0.47	0.47	0.80
Ile36	57.5	39.7	26.9	17.1	13.4	1.31	0.86	0.69	0.56
Ile44	58.7	42.4	27.7	16.8	13.7	1.41	0.76	0.56	0.24
Ile61	61.7	36.5	27.5	16.0	13.8	1.31	0.23	0.30	0.33

a) All of the ^{13}C and ^{1}H chemical shifts were obtained by ^{1}H-detected 3D ^{13}C/^{13}C/^{1}H correlation experiments in Fig. 3. Once the connectivity of the signals were established, the signals were assigned to each Ile residue based on $^{13}C_\alpha$ and $^{13}C_\beta$ assignments in previous SSNMR studies of microcrystalline human ubiquitin.[4, 5] All chemical shifts were calibrated based on DSS standard.

b) Not observed presumably for dynamics and/or scrambling.

Supplementary References:

1. Fung BM, Khitrin AK, Ermolaev K. An improved broadband decoupling sequence for liquid crystals and solids. Journal of Magnetic Resonance. 2000;142(1):97-101.
2. Shaka AJ, Keeler J, Frenkiel T, Freeman J. An improved sequence for broadband decoupling: WALTZ-16. J Magn Reson. 1983;52:335-338.
3. Ishii Y, Tycko R. Sensitivity enhancement in solid state 15N NMR by indirect detection with high-speed magic angle spinning. Journal of Magnetic Resonance. 2000;142:199-204.
4. Igumenova TI, McDermott AE, Zilm KW, Martin RW, Paulson EK, Wand AJ. Assignments of carbon NMR resonances for microcrystalline ubiquitin. Journal of the American Chemical Society. 2004;126(21):6720-6727.
5. Manolikas T, Herrmann T, Meier BH. Protein structure determination from C-13 spin-diffusion solid-state NMR spectroscopy. Journal of the American Chemical Society. 2008;130(12):3959-3966.
